# Supplementary material for: Peer-Education as a Tool to Educate on Antibiotics, Resistance and Use in 16–18-Year-Olds: A Feasibility Study
Source: Antibiotics (Basel). 2020 Mar 30;9(4):146. doi: 10.3390/antibiotics9040146 (PMC7235882; doi:10.3390/antibiotics9040146)
Supplement: Supplementary file 1 [file antibiotics-09-00146-s001.zip › File S1 - Antibiotic_PE_Complete pack FINAL v5 13.10.17.pdf]

e-Bug
Young Adult

Antibiotics Peer Education

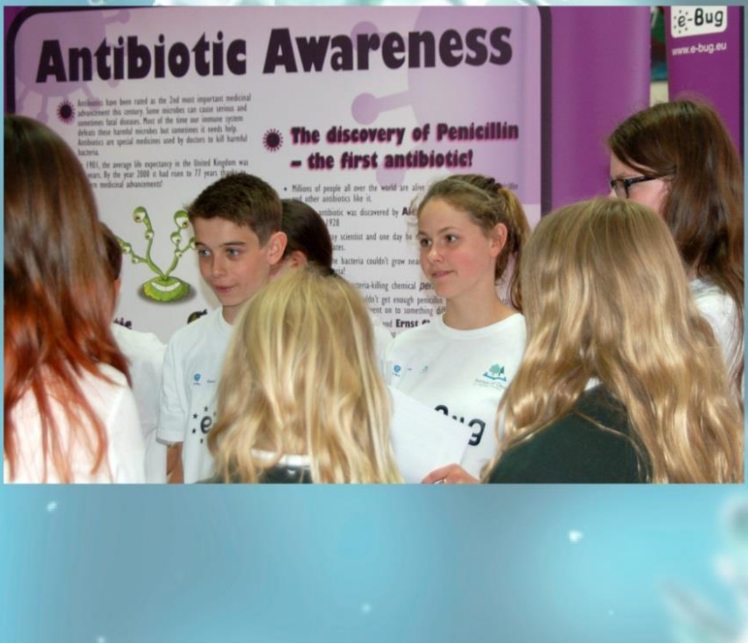

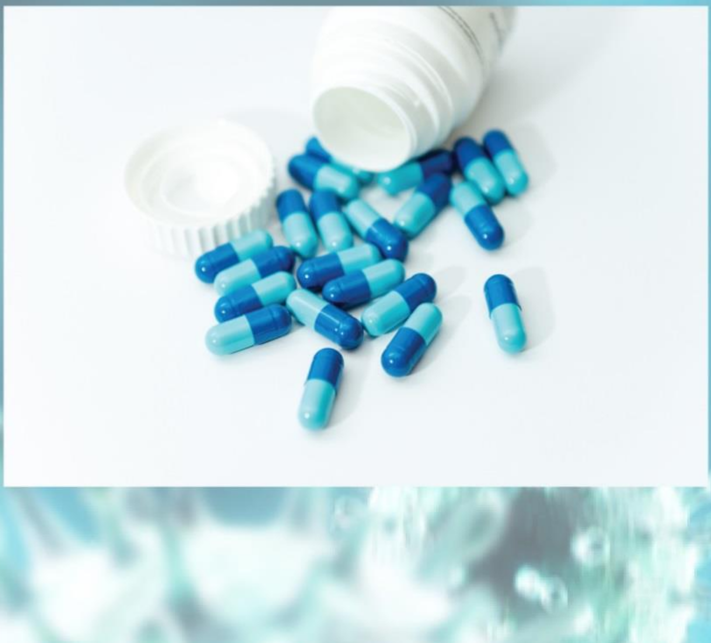

Key Stage 4&5 / Science*

*Certain sections may also link with the PSHE curriculum

Introduction

Within this lesson plan, students aged 16-18 years will run a 1 hour lesson on antibiotics and antibiotic resistance with other young people.

Information for Teachers

Value of peer education

Peer education is becoming an increasingly popular educational tool due to the benefits for all involved. For the peer educators, benefits can include positive changes in knowledge, skills, attitudes and confidence, and development of key communication and social skills. By teaching others, students gain a deeper understanding of the topics covered, and have increased knowledge in the area, when compared to didactic learning.

Students taught by their peers may identify more closely with their educator, which allows the development of positive relationships and a greater level of trust between educator and student.

What is covered?

Within this lesson plan, all students will cover the important topics of antibiotics and antibiotic resistance. Not only will students learn the science behind how antibiotics work and how resistance to antibiotics comes about, they will also learn essential health information, such as how to take antibiotics correctly, which is important for PSHE education.

Running the lesson

The lesson can be arranged to suit any educational establishment. The lesson set-up will be flexible, in that some lessons may be incorporated into an assembly, some may be used in single lessons, or groups of classes may be taught together. The peer educators, who may be university students or 16-18 year olds, should work in small teams, of between 2 and 6 students to deliver the lesson, deciding between themselves how to divide up the lesson delivery.

The peer educators should ensure that they cover the same learning outcomes and activities across all lessons.

Peer educators should be allowed the time to prepare and practice before the lesson delivery.

Antibiotics: Peer Education

Lesson Plan

National Curriculum / exam specification links

Key Stage 5 (age 16-18):

This lesson plan covers several topics found in the AQA, OCR, Edexcel and WJEC examination specification for Biology, Human Biology and related subjects.

More information can be found on our 'Examination Links' webpage.

Aim of the lesson

- To teach young people how to use antibiotics responsibly and that overuse will increase antibiotic resistance

Learning outcomes

- Many infections get better on their own without the need for antibiotics
- Bacterial and viral infections may cause similar symptoms
- Antibiotics work on bacteria and have no effect on viruses
- Bacteria are continually adapting to develop ways of not being killed by antibiotics (known as antibiotic resistance)
- Antibiotic resistance can spread between different bacteria in our body
- Antibiotics can affect all the bacteria in your body, not just the ones which cause an infection
- Antibiotic resistant bacteria can be carried by healthy or ill people and passed on silently to others
- The more often you take antibiotics, the more likely you are to have an antibiotic resistant infection
- You should not share antibiotics as each antibiotic is personal to you and your infection
- Antibiotics should always be taken as instructed by a doctor or nurse, because overuse may make the antibiotics less effective against the bacteria, and then the next time we have an infection they may not work
- Examples of antibiotics include amoxicillin for chest infections, flucloxacillin for skin infections, and nitrofurantoin for urine infections.
- Painkillers such as paracetamol, ibuprofen, aspirin and codeine are NOT antibiotics.

Background information for Peer Educators

Antibiotics are special medicines which can only be prescribed by a doctor or nurse. Antibiotics are used to treat bacterial infections such as meningitis, tuberculosis and pneumonia. They do not work on viruses, so antibiotics cannot treat viral infections such as colds and flu. Penicillin was the first antibiotic to be discovered in 1928 by Alexander Fleming and is still used to treat some sore throats and pneumonia today. Other examples of antibiotics include amoxicillin for chest infections, flucloxacillin for skin infections, and nitrofurantoin for urine infections. Painkillers such as paracetamol, ibuprofen, aspirin and codeine are NOT antibiotics.

Antibiotics can be broad spectrum, affecting many different species of bacteria, or narrow spectrum, affecting only one or two. Antibiotics work by targeting structures unique to bacteria, so they are not dangerous to human cells and they do not kill viruses. Targets include the bacterial peptidoglycan cell wall, the ribosome (needed for protein production), DNA replication (needed for cell division) and metabolic enzyme activity (needed for cell growth).

Bacteria are continually adapting to develop ways of not being killed by antibiotics. This is called antibiotic resistance. Resistance develops due to a change in the bacterial DNA. These genes for antibiotic resistance can then spread between different bacteria in our bodies. Antibiotic resistant bacteria can be carried by healthy or ill people and can spread to others just as other types of microbes would, for example via hands or by touching surfaces where bacteria are present.

Antibiotic resistance arises due to the overuse and misuse of antibiotics. The more often a person takes antibiotics, the more likely they are to develop antibiotic resistant bacteria in their body. To prevent resistance, antibiotics should only be taken as prescribed by a doctor or nurse.

The important points to remember are:

1. Many infections get better on their own, without the need for antibiotics.
2. Antibiotics should only be taken for bacterial infections and not viral infections such as colds and flu, and most coughs, sore throats, ear infections or sinusitis.
3. It is important to take antibiotics exactly as instructed (for example three times daily), to ensure all bacteria within your body are killed and to prevent the development of antibiotic resistance.
4. Antibiotics are personal and prescribed for individuals and for a particular infection. They should not be shared or taken for a different illness.

Section 1: Introducing Antibiotics and Antibiotic Resistance (20-25 mins)

Introducing antibiotics

Materials required: Microbe fact sheet on the three types of microbes and their relative sizes – available here (Microbe Fact Sheet SH1).

- Begin by asking the students if they know three types of microbes that can cause infections – bacteria, virus and fungi, and explain the relative sizes of the microbes (fungi are the largest and viruses are the smallest). Explain that infections are treated differently depending on the microbe that has caused it.
- Introduce antibiotics – ask who has heard of them and if anyone knows which microbe they affect. Explain that antibiotics only affect bacteria, and will have no effect on viruses. Give examples of antibiotics - amoxicillin for chest infections, flucloxacillin for skin infections, and nitrofurantoin for urine infections. Explain that painkillers, both ones that are prescribed and ones you can buy over the counter are NOT antibiotics. Provide examples of painkillers - paracetamol, ibuprofen, aspirin and codeine.
- Ask the students if they know of any illnesses caused by viruses. Examples you can give include colds and flu, chickenpox, measles, mumps and rubella. Ask the students if they know of any illnesses caused by bacterial infections. Examples you can give include pneumonia, meningitis, whooping cough, chlamydia, and urinary tract infections.
- Ask the students, if it is easy to tell the difference between bacterial and viral infections. How should viral infections be treated? It can be explained that rest and fluids can help with viral infections, and taking paracetamol for pain relief.
- It is also important to say that many bacterial infections get better on their own without antibiotics.
- Show the students the Antibiotic Guardian video, available at http://antibioticguardian.com. The clip can be used to stimulate a discussion between the students.

Optional: The students can be asked at the end of the lesson to become an Antibiotic Guardian by pledging to use antibiotics responsibly.

Antibiotic Resistance

Materials Required: Antibiotic resistance presentation – available here

Optional preparation: Research information on MRSA and TB. Information can be found on the Public Health England, NHS Choices, MRSA Action UK and the Stop TB Partnership websites.

Core task: Show students the antibiotic resistance presentation available on the senior student e-Bug website about the discovery of antibiotics and antibiotic resistance. Introduce antibiotic resistance by explaining that bacteria are continually developing ways to avoid being killed by antibiotics, and that this is known as antibiotic resistance. Antibiotic resistant bacteria can be very dangerous as they cannot be treated.

Optional task: Ask if anyone has heard of MRSA? Describe MRSA and antibiotic resistant TB.

Key points to cover in this section:

- Relative sizes of microbes: fungi are the largest, followed by bacteria, and then viruses.
- Many bacterial infections get better on their own without the need for antibiotics.
- It is not easy to tell the difference between viruses and bacteria because they can have similar symptoms including coughing and sneezing, diarrhoea, fever, and fatigue and vomiting.
- Viral infections can be treated with rest, drinking fluids, taking painkillers, and eating healthily.
- Antibiotics work on bacteria and have no effect on viruses.
- Bacteria are continually adapting to develop ways of not being killed by antibiotics (known as antibiotic resistance).

Key words to use in this section:

- Bacteria
- Virus
- Fungi
- Microbe
- Antibiotics
- Antibiotic resistance
- Infection
- Symptom

Section 2: Antibiotic Resistance activities (30 – 40 mins)

Activity 1: Demonstrating antibiotic resistance using balloons

Now explain that you will use a demonstration to describe antibiotic resistance.

Materials required: Balloons, sellotape or parcel tape, pin, glitter (optional)

Preparation:

- Blow up 4 balloons in one colour and 2 balloons in another colour (yellow and red are used here to describe the demonstration).
- Add a strip of sellotape or parcel tape to the top of the two balloons which are a different colour. Clear parcel tape works the best; if sellotape or brown parcel tape is used, several layers may be required for the experiment to work. The sellotape is best placed on the top of the balloon where the balloon is thickest.

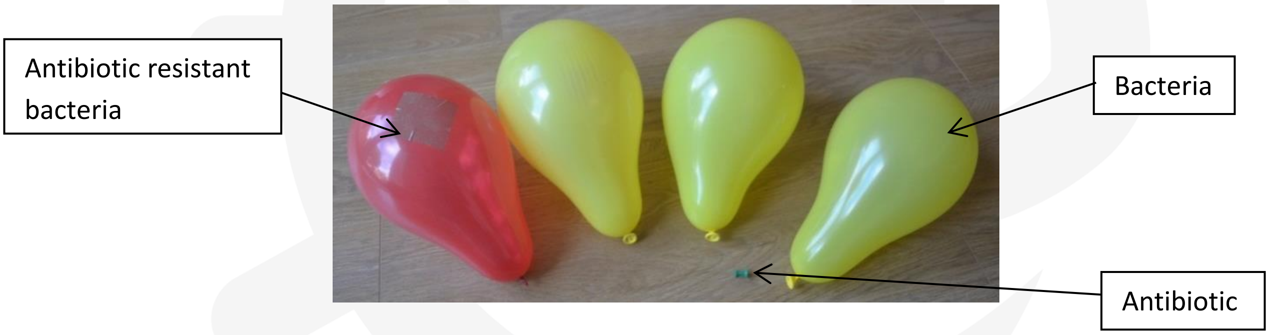

Brown tape is used for demonstration but clear parcel tape is ideal to use as resistant is carried slightly/invisibly in people who are not ill**.**

Activity:

- Explain to the students that the yellow balloons represent bacteria and the red balloon with tape on represents **antibiotic resistant** bacteria. The pin represents the antibiotic.
- When we give an antibiotic, bacteria are killed or damaged – pop some yellow balloons with the pin.
- If you put glitter in the balloon it also demonstrates that viruses are not killed by antibiotics and can continue to spread.
- In particular, one group of antibiotics (the penicillins) damage the bacterial cell wall. However in bacteria that are antibiotic resistant, the cell walls are now not affected by the antibiotics – put the pin through the sellotape in the red balloons, it will not pop.

Antibiotic resistant bacteria are not killed by antibiotic

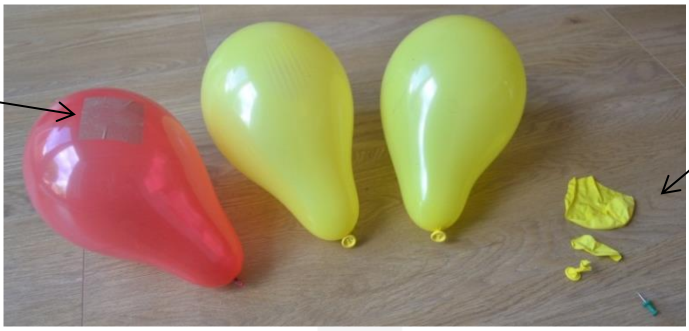

Bacteria killed by antibiotic

This makes it more likely for the resistant bacteria to survive and reproduce. They have a selective advantage.

- Ask if anyone knows where resistance comes from. Explain it is due to a change in the bacterial DNA/genes that tell the bacteria how to make the cell wall or enzyme.
- Explain that bacteria can pass these resistant genes on to other bacteria – put sellotape on a remaining yellow balloon, which represents the transfer of antibiotic resistance to another bacterium. This can happen in our bodies.

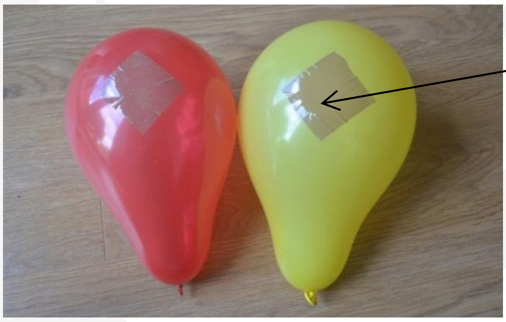

Antibiotic resistance can spread to other bacteria

- Resistance is also passed on when bacteria reproduce – demonstrate this by blowing up another red balloon and putting sellotape on it.

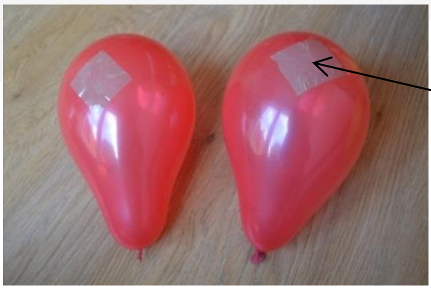

Antibiotic resistance is passed on when bacteria reproduce

- Explain that resistant bacteria can be passed on from person to person just as normal bacteria can. Ask how these bacteria can spread? The easiest way is via our hands. Examples include direct skin to skin contact or touching surfaces which may contain bacteria.

To prevent bacteria becoming resistant to antibiotics, we should always take antibiotics correctly, as the doctor or nurse prescribes. The more often we take antibiotics, the more likely we are to have antibiotic resistant bacteria in our bodies. Therefore overusing antibiotics may make them less effective.

- Ask if anyone knows what we mean by responsible use of antibiotics. Correct responses can include: not taking them for a cold or flu, not sharing antibiotics, not taking other people's antibiotics or leftovers, taking the course as prescribed by a doctor or nurse, and not taking them for longer than necessary.

Students will then carry out activities 2a and 2b below to learn how to take antibiotics correctly.

Activity 2a: Antibiotic scenarios

Materials required: Student scenarios – available in this booklet (student handout1), Managing Your Infection leaflet available here. Print this out in A5 so that it can be folded up as a leaflet.

The scenarios in student handout 1 can be used to teach about how to take antibiotics correctly.

- Give each student a copy of the worksheets. Students can work in small groups for this activity. The worksheets have three scenarios which teach the group not to take antibiotics for colds and flu, to take antibiotics as prescribed and not to use other people's or leftover antibiotics.
- Ask the students to use the Managing Your Infection leaflet to help them think of possible correct and incorrect answers. Discuss these for each of the scenarios. An answer sheet is provided to aid discussions.
- Ask the students to take the Managing Your Infection leaflet home to discuss with their family.

Activity 2b: Antibiotics "Right" or "Wrong"?

Materials required: 'Right or Wrong' worksheet – available in this booklet (student handout 2).

- Give each student a copy of the worksheet (student handout 2). The worksheet has 8 statements, which teaches the students how to take antibiotics correctly. Students can work in small groups for this activity and for each statement, discuss with the group whether they are right or wrong, and reasons why. An answer sheet is provided to aid discussions.

Alternative options for delivery include printing each speech bubble on a separate piece of paper, laminating and discussing with the group. Alternatively, each speech bubble can be included on a different slide in a PowerPoint presentation.

Key points to cover in this section:

- Antibiotic resistance can spread between different bacteria in our body.
- Antibiotics can affect both good and bad bacteria in our bodies, not just the ones which cause an infection.
- Antibiotic resistant bacteria can be carried by healthy or ill people and passed on silently to others.
- Resistance can be spread by reproduction of antibiotic resistant bacteria or by resistance spreading from one bacterium to another.
- The more often you take antibiotics, the more likely you are to have an antibiotic resistant infection.
- You should not share antibiotics as each antibiotic is personal to you and your infection.
- Antibiotics should always be taken as instructed by a doctor or nurse, because overuse may make the antibiotics less effective against the bacteria, and then the next time we have an infection they may not work.

Key words to use in this section:

- Antibiotic
- Antibiotic resistance
- Bacterial cell wall
- Overuse
- Spread of infection

Recap (5 mins)

Recap the lesson and provide students with a summary of the 3 main points to take away from the lesson:

1. Summarise what antibiotics are used for (bacterial infections).
2. How antibiotics should be taken responsibly (not taking them for a cold or flu, not sharing antibiotics, not taking other people's antibiotics or leftovers, taking the course as prescribed by a doctor or nurse, not taking them for longer than necessary.
3. Summarise why antibiotics should not be overused (increase in antibiotic resistance).

Remind students to take the Managing Your Infection leaflet home to share with family members.

Activity 2a: Antibiotic Scenarios

Scenario 1: Ash has a runny nose and a really sore throat so he goes to the doctor.

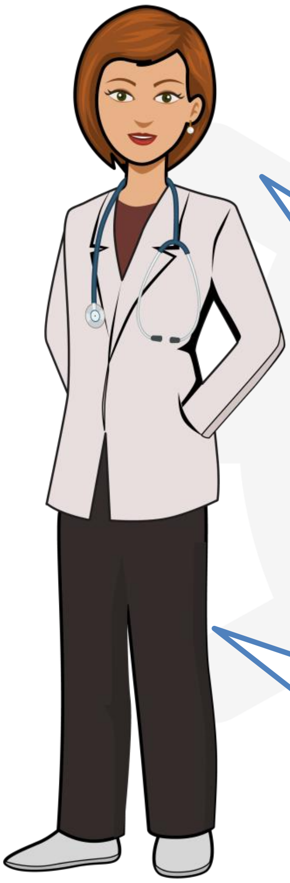

Doctor: A runny nose isn't helped by antibiotics. Go home and go to bed, and take some painkillers for your sore throat.

Ash: But I'm really ill. Surely antibiotics will help?

Doctor: Antibiotics don't work for everything. Bacterial and viral infections can make you feel really bad. Sore throats can last a week.

Ash: What if I get really bad?

Doctor: The pharmacist will be able to give you something for your pain.

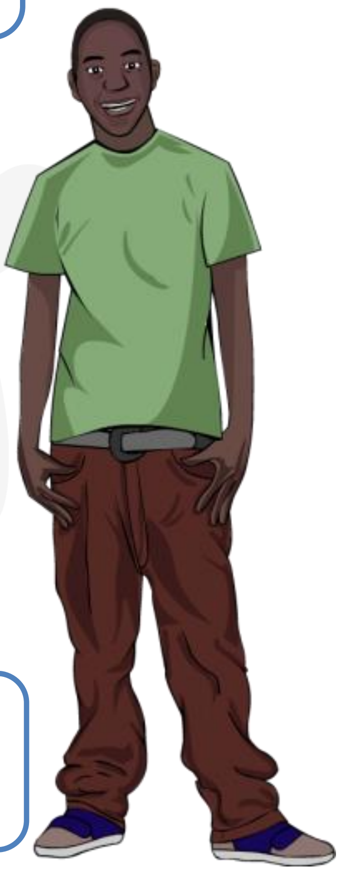

DISCUSSION

Discuss whether you agree or disagree with Ash going to the pharmacy?

Discuss what you think Ash might be worried about?

Activity 2a: Antibiotic Scenarios

Scenario 2: Alisha is talking to her friend Anna about her urine infection. Alisha has been prescribed antibiotics by her doctor.

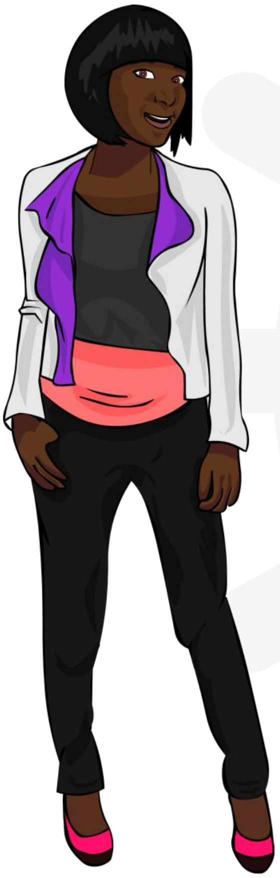

Alisha: I've been given antibiotics for my urine infection. The doctor told me to take them for 3 days, morning and evening. I've taken them for 3 days, but I've got some extra, what shall I do with the extra ones now I feel better?

Anna: Why don't you just take them, it will make sure you get rid of all the infection.

DISCUSSION

Discuss whether you agree or disagree with Anna?

Discuss what you think Alisha should do with her extra antibiotics?

Activity 2a: Antibiotic Scenarios

Scenario 3: Chloe was talking to her friend Jamie about her headache and cough.

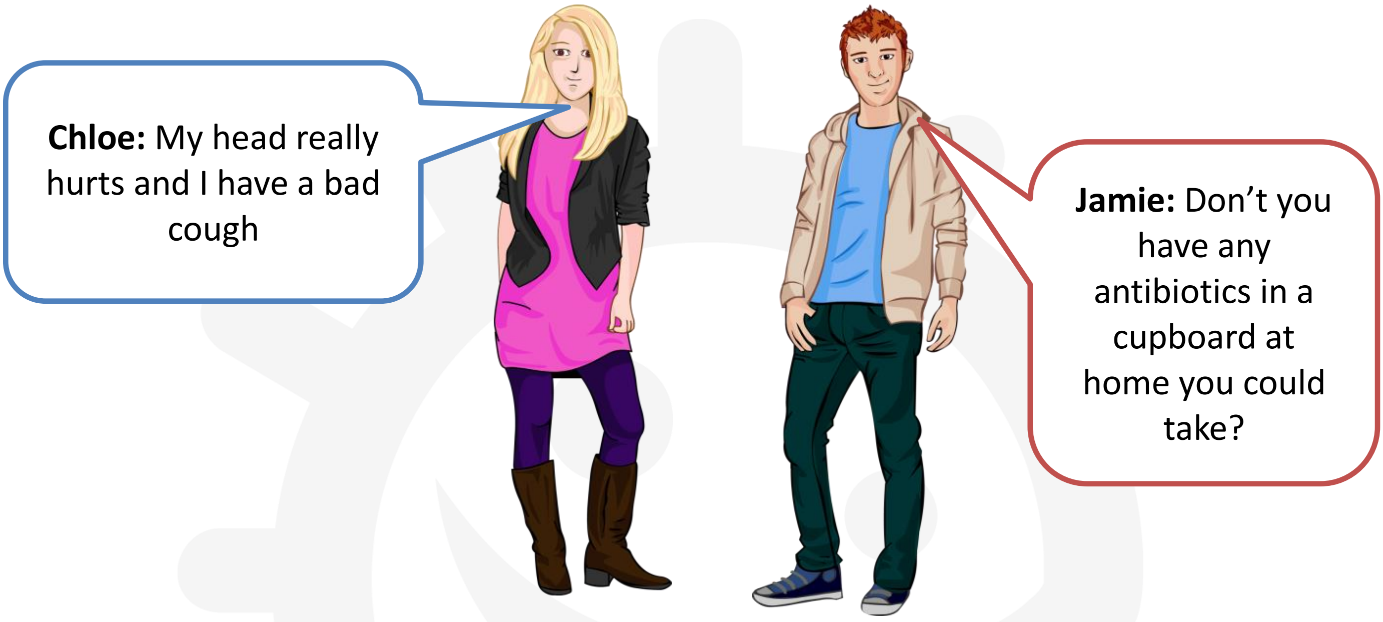

What should Chloe say? Discuss the correct and incorrect options.

Statement	Correct	Incorrect
Gosh no, I shouldn't take anyone else's antibiotics.		
Great idea – we have some left over from when my sister had an ear infection.		
Yes I had a cough a few weeks ago and went to the out of hours and they gave me a prescription, but I didn't bother to cash it in. I'll do it now!		
I don't have any antibiotics in the cupboard – don't you always take them back to the pharmacy?		
I had a urine infection last month. I'll use the leftover antibiotics from them.		
I shouldn't take antibiotics that are left over.		
I don't have any at home, but I'll ask Josie, she is always at the doctor with her coughs.		
I only take antibiotics if the doctor prescribes them, otherwise I might get antibiotic resistant bugs.		
I think I should just take some pain relief and go to bed.		

Activity 2a: Antibiotic Scenarios – answer sheet

Scenario 1

Ash had a runny nose and a really sore throat so he went to the doctor

Discussion points:

- Many infections get better on their own without the need for antibiotics.
- Antibiotics won't make a difference to how long your symptoms/illness/infection lasts.
- All runny noses are caused by viruses which antibiotics do not work on – so there is no point having an antibiotic.
- Paracetamol is really useful to take for the pain.
- If you become ill very quickly, have a really red throat or pus on your tonsils with high temperature and no cough or runny nose, you are more likely to benefit from antibiotics.

Scenario 2

Alisha has a urine infection and has been prescribed antibiotics by her doctor

Discussion points:

Correct options:-

- Do what the doctor says and take them for the 3 days.
- Take the leftovers back to the pharmacy (do not flush antibiotics down the toilet otherwise antibiotics get into the water system).
- If you take more days, your bugs are more likely to become resistant, and the antibiotics are less likely to kill the bacteria the next time you need them.
- Don't take any extras as that will kill more of your useful bugs in your gut too, causing diarrhoea or thrush (overgrowth of fungi).

Incorrect options if needed:-

- Keep the rest for next time.
- Stop taking them now. What's the point in taking them if you are feeling better?
- Take the whole pack and then you'll definitely kill the bugs.

Scenario 3

Chloe was talking to her friend Jamie about her headache and cough

	Correct	Incorrect
Gosh no, I shouldn't take anyone else's antibiotics.	✓	
Great idea – we have some left over from when my sister had an ear infection.		✓
Yes I had a cough a few weeks ago and went to the out of hours and they gave me a prescription, but I didn't bother to cash it in. I'll do it now!		✓
I don't have any antibiotics in the cupboard – don't you always take them back to the pharmacy?	✓	
I had a urine infection last month. I'll use the leftover antibiotics from them.		✓
I shouldn't take antibiotics that are left over.	✓	
I don't have any at home, but I'll ask Josie, she is always at the doctor with her coughs.		✓
I only take antibiotics if the doctor prescribes them, otherwise I might get antibiotic resistant bugs.	✓	
I think I should just take some pain relief and go to bed.	✓	

Discussion points:

- If you have taken an antibiotic in the last 6 months, a bacterial infection is twice as likely to be antibiotic resistant.
- Repeated courses of antibiotics are associated with a greater risk of antibiotic resistant bacteria.
- Antibiotics are specific for each infection, for example an antibiotic for a sore throat may not work for a urine infection and vice versa, so you should never share others antibiotics or leftovers.
- Pain relief such as paracetamol help cold, flu, sore throat and earache symptoms and help bring down a temperature.
- The dose of antibiotic is specifically chosen for each infection, so that the antibiotic reaches the infection and kills the bacteria.

Learning outcome checklist

- Relative sizes of microbes: fungi are the largest, followed by bacteria, and then viruses.
- Many bacterial infections get better on their own without the need for antibiotics.
- It is not easy to tell the difference between viruses and bacteria because they can have similar symptoms including coughing and sneezing, diarrhoea, fever, and fatigue and vomiting.
- Viral infections can be treated with rest, drinking fluids, taking painkillers, and eating healthily.
- Antibiotics work on bacteria and have no effect on viruses.
- Bacteria are continually adapting to develop ways of not being killed by antibiotics (known as antibiotic resistance).
- Antibiotic resistance can spread between different bacteria in our body.
- Antibiotics can affect both good and bad bacteria in our bodies, not just the ones which cause an infection.
- Antibiotic resistant bacteria can be carried by healthy or ill people and passed on silently to others.
- Resistance can be spread by reproduction of antibiotic resistant bacteria or by resistance spreading from one bacterium to another.
- The more often you take antibiotics, the more likely you are to have an antibiotic resistant infection.
- You should not share antibiotics as each antibiotic is personal to you and your infection.
- Antibiotics should always be taken as instructed by a doctor or nurse, because overuse may make the antibiotics less effective against the bacteria, and then the next time we have an infection they may not work.

Activity 2b: Antibiotics Right or Wrong?

Discuss: Which of these statements are right or wrong?

1. He was coughing and sneezing everywhere. You would have thought the doctor would have given him antibiotics!

2. You can't have antibiotic resistant bacteria you are too healthy, and don't have any illness or infection!

3. When my friend was ill, I gave her my old antibiotics. I like helping my friends.

4. Antibiotics don't help colds and flu; you just need bed rest, lots of fluids, pain relief and to eat healthily.

5. All drugs are bad for you. I can't see the point in taking antibiotics.

6. My doctor gave me 10 days antibiotics for my tonsillitis, but I feel better after 3 days so I'm going to stop taking them.

7. Rubbish, antibiotics don't give you diarrhoea, - they can't affect the bacteria in your gut!

8. The more I take antibiotics the more likely I am to get an antibiotic resistant infection.

Educator Sheet

Statement	Right or Wrong	Reason
**He was coughing and sneezing everywhere. You would have thought the doctor would have given him antibiotics!**	Wrong	Most common infections will get better by themselves through time, bed rest, liquid intake and healthy living. Antibiotics do not work on viruses
**You can't have antibiotic resistant bacteria, you are too healthy, and don't have any illness or infection**	Wrong	Everyone carries at least a few bacteria that are not killed by antibiotics and these are called antibiotic resistant bacteria. When you take antibiotics, these resistant bacteria multiply more than the others, and can also spread their resistance genes to other bacteria
**When my friend was ill, I gave her my old antibiotics. I like helping my friends**	Wrong	You must not use other people's antibiotics or any leftover antibiotics
**Antibiotics don't help colds and flu; you just need bed rest, lots of fluids, pain relief and to eat healthily**	Right	Most common infections will get better by themselves through time, bed rest, liquid intake and healthy living. Antibiotics do not work on viruses
**All drugs are bad for you. I can't see the point in taking antibiotics**	Wrong	Antibiotics can help severe infections such as meningitis, pneumonia or kidney/urine infections
**My doctor gave me 10 days antibiotics for my tonsillitis, but I feel better after 3 days so I'm going to stop taking them**	Wrong	Take antibiotics exactly as given by your doctor or nurse. Even if you feel better after 3 days you might still have the infection
**Rubbish, antibiotics don't give you diarrhoea, - they can't affect the bacteria in your gut!**	Wrong	Most common infections like the flu will get better by themselves through time, bed rest, liquid intake and healthy living. Antibiotics do not work on headaches you get with colds and flu or viruses
**The more I take antibiotics the more likely I am to get an antibiotic resistant infection**	Right	If you overuse antibiotics they might not work when you really need them for a severe infection

In this pack you will find e-Bug peer education activities for lessons on antibiotics, designed for young adults to deliver to their peers. The activities focus on antibiotics, antibiotic resistance and practical advice on how to take antibiotics correctly.

Background information and full instructions are provided for peer educators on how to deliver the lesson, as well as learning outcomes the activities covered. It is recommended peer educators have enough time to prepare and undertake the necessary background research before delivering a lesson.

We hope you enjoy using this lesson pack to help teach and inspire students.
