# Supplementary material for: Peer-Education as a Tool to Educate on Antibiotics, Resistance and Use in 16–18-Year-Olds: A Feasibility Study
Source: Antibiotics (Basel). 2020 Mar 30;9(4):146. doi: 10.3390/antibiotics9040146 (PMC7235882; doi:10.3390/antibiotics9040146)
Supplement: Supplementary file 1 [file antibiotics-09-00146-s001.zip › File S2 - PRE- Knowledge questionnaire _ FINAL_08.11.2017.docx]

| 1. **Which of these microbes cause colds and flu?**   **Antibiotic Pre- Questionnaire**  **Please tick only ONE box for each question** | | 1. **To treat colds and flu we should:** | | |
| --- | --- | --- | --- | --- |
| `Bacteria |  | Take antibiotics | |  |
| Viruses |  | Rest, drink plenty of fluids and take pain relief | |  |
| Fungi |  | Have the flu vaccination | |  |
| None of the above |  | Visit the hospital | |  |
|  |  |  | |  |
| 1. **Which of the following is an antibiotic?** |  |  | |  |
| Aspirin |  |  |  | |
| Paracetamol |  |  |  | |
| Flucloxacillin |  |  |  | |
| Codeine |  |  |  | |
|  |  |  | |  |
| 1. **Bacterial and viral infections can cause similar symptoms**   **Name:**  **Date of birth:** | | 1. **Antibiotics help cure colds** | | |
| True – they can both make you feel really bad |  | True – they are the most effective form of treatment | |  |
| False – symptoms are different if you have a virus |  | False – colds are caused by viruses which are not affected by antibiotics | |  |
| True – bacteria are a type of virus, they do the same thing |  | False – antibiotics could make a cold worse | |  |
| False – you will feel much worse if you have a virus |  | True – but only as a last resort | |  |
|  |  |  | |  |
| 1. **Antibiotics kill:** |  | 1. **Antibiotics can kill good bacteria in the gut leading to diarrhoea** | | |
| Viruses only |  | True – they kill both good and bad bacteria | |  |
| Fungi only |  | False – they are only designed to target bad bacteria | |  |
| Bacteria only |  | True – the gut only contains good bacteria | |  |
| Both bacteria and viruses |  | False – antibiotics only work on microbes that live in your respiratory system | |  |
|  |  |  | |  |
| 1. **When taking antibiotics you should:** | | 1. **Taking antibiotics when they are not needed can lead to:** | | |
| Take them for more days than you need to, to make sure the infection doesn’t come back |  | A weaker immune system to help you fight off infection | |  |
| Keep some for the next time you are ill |  | Antibiotics working faster next time you are ill | |  |
| Take the antibiotics as instructed by a doctor or nurse |  | Long-term protection from bacterial infections | |  |
| Share them with your friends if they are unwell |  | Bacteria becoming resistant, so next time you have an infection they may not work | |  |
|  | | | |  |
| 1. **Antibiotic resistance is:** | | 1. **Antibiotic resistance is caused by:** | | |
| When bacteria develop ways to avoid being killed by antibiotics |  | Taking painkillers such as paracetamol or ibuprofen | |  |
| When viruses develop ways to avoid being killed by antibiotics |  | The overuse of antibiotics | |  |
| When antibiotics are no longer able to kill viruses |  | Vaccinations | |  |
| When people become resistant to antibiotics |  | Alternative medicines | |  |

| 1. **How do antibiotic resistant bacteria spread?** | | 1. **Healthy people can carry antibiotic resistant bacteria and pass this on to others** | |
| --- | --- | --- | --- |
| Resistant bacteria pass on resistant genes to other bacteria |  | True – both healthy and ill people can carry and pass on antibiotic resistant bacteria |  |
| Reproduction of resistant bacteria |  | False – only ill people carry and pass on antibiotic resistant bacteria |  |
| From person to person |  | False – you cannot be healthy if you have antibiotic resistant bacteria |  |
| All of the above |  | False – you can’t pass on your own bacteria to others |  |

**Antibiotics Scenarios**

| 1. **Liam has a sore throat and a headache. What can Liam do to help him get better? Pick the best option** | |
| --- | --- |
| Visit the doctor to pick up a prescription for antibiotics |  |
| Take some of his mum’s leftover antibiotics |  |
| Rest and drink plenty of fluids |  |
| Get plenty of exercise |  |
|  |  |
| 1. **Anisha has been given antibiotics by the doctor for a urine infection. She has been prescribed them for three days, but starts to feel better after one day. Anisha stops taking them. Should she have stopped? Pick the best option** | |
| Yes – taking too many antibiotics is bad for you, it is a good idea she stopped taking them early |  |
| No – only taking the antibiotics for one day will mean the infection is more likely to come back or become antibiotic resistant |  |
| Yes – but if she had another type of infection she should keep taking them |  |
| No – you should always take as many antibiotics as possible |  |

| 1. **Louise does not like to see her friend Chris unwell. Chris has a cold and a dry cough. She finds some of her leftover antibiotics in the cupboard and asks Chris if he wants to use them. What should Chris say to Louise? Pick the best option** | |
| --- | --- |
| ‘Thanks Louise, you’re a really good friend. I’ll take these, then hopefully I will feel better by the weekend’ |  |
| ‘I’m not sure if my cough is really that bad. I’ll take only one tablet, just in case’ |  |
| It’s just a cold virus Louise, so antibiotics won’t work. It would be a good idea to return any old antibiotics to the pharmacy’ |  |
| ‘It’s okay Louise; I’ve got an appointment at the doctor’s tomorrow, so I should get some antibiotics then’ |  |

**Thank you for completing this questionnaire**
